# Antimicrobial activity and enterococcus faecalis biofilm formation on chlorhexidine varnishes

**DOI:** 10.4317/medoral.17680

**Published:** 2012-02-09

**Authors:** María T. Arias-Moliz, Carmen M. Ferrer-Luque, María P. González-Rodríguez, Esther Navarro-Escobar, Márcia F.A. de Freitas, Pilar Baca

**Affiliations:** 1 DDS, PhD, Assistant Professor, Department of Microbiology, School of Dentistry. University of Granada, Campus de Cartuja, Colegio Máximo s/n, Granada, Spain; 2 DDS, MD, PhD, Associate Professor Department of Preventive and Operative Dentistry, School of Dentistry. University of Granada, Campus de Cartuja, Colegio Máximo s/n, Granada, Spain; 3 DDS, PhD, Associate Professor, Department of Preventive and Operative Dentistry, School of Dentistry. University of Granada, Campus de Cartuja, Colegio Máximo s/n, Granada, Spain; 4 BDS, Postgraduate Student, Department of Preventive and Operative Dentistry, School of Dentistry. University of Granada, Campus de Cartuja, Colegio Máximo s/n, Granada, Spain; 5 DDS, Postgraduate Student, Department of Dental Materials, School of Dentistry. University of Sao Paulo, Rua Barão do Triunfo, Sao Paulo, Brazil; 6 DDS, MD, PhD: Professor, Department of Preventive and Operative Dentistry, School of Dentistry. University of Granada, Campus de Cartuja, Colegio Máximo s/n, Granada, Spain

## Abstract

Objective: To evaluate, in vitro, the antimicrobial activity and biofilm formation of three chlorhexidine varnishes in four Enterococcus faecalis strains: E. faecalis ATCC 29212, E. faecalis EF-D1 (from failed endodontic treatment), E. faecalis 072 (cheese) and E. faecalis U-1765 (nosocomial infection), and one Enterococcus durans strain (failed endodontic treatment). Study Design: The direct contact test was used to study the antimicrobial activity. Bacterial suspensions were exposed for one hour to EC40, Cervitec (CE) and Cervitec Plus (CEP) varnishes. “Eradication” was defined as 100% bacterial kill. The formation of enterococci biofilms was tested on the surface of the varnishes after 24 hours of incubation and expressed as percentage of biofilm reduction. Results: EC40 eradicated all strains except E. faecalis ATCC 29212, where 98.78% kill was achieved. CE and CEP showed antimicrobial activity against all the strains, but most clearly against E. durans and E. faecalis 072. EC40 completely inhibited the formation of biofilm of E. faecalis ATCC 29212, E. faecalis 072 and E. durans. CE and CEP led to over 92% of biofilm reduction, except in the case of E. faecalis U-1765 on CEP (76.42%). Conclusion: The three varnishes studied were seen to be effective in killing the tested strains of enterococci and in inhibiting the formation of biofilm, the best results being observed with EC40.

** Key words:**Biofilm, chlorhexidine varnish, direct contact test, Enterococcus durans, Enterococcus faecalis, intracanal medication.

## Introduction

An important aim of endodontic therapy is the elimination of microorganisms from root canal systems. This may be accomplished using mechanical instrumentation and chemical irrigation, and intracanal medication for persistent infections (1).

Chlorhexidine (CHX) is a cationic molecule with a wide antimicrobial spectrum against both Gram positive and Gram negative bacteria (2). These would include *Enterococcus faecalis* (3), a microorganism frequently isolated from necrotic and failed endodontic treatments (4). Its antibacterial activity and substantivity (5) make CHX widely used as a root canal irrigant (6,7) and intracanal medication (8) and it is known to reduce and/or delay the entry of bacteria into the root canal system (9).

Available in different formulations, CHX is commonly used in endodontics as a solution or gel (2). CHX has recently been incorporated into a variety of sustained-release systems, including varnishes, in order to prolong the period of active-agent delivery and achieve maximum antimicrobial effectiveness (10). Some of these slow-release systems have proven effective as intracanal medication against *E. faecalis* (11-13). The CHX varnishes apparently penetrate and seal tubules in dentin (14), reducing the level of mutans streptococci in exposed root surfaces (15) and it may help control established root caries lesions (16). However, its antimicrobial activity against bacteria involved in root canal infections is not known. The aim of this study was therefore to evaluate, in vitro, the antimicrobial activity and the ability to reduce biofilm formation of three CHX varnishes, two of them containing thymol, against four *E. faecalis* strains and one *E. durans* strain.

## Material and Methods

-Microorganisms and tested materials

The bacterial strains used in the study and their source are listed in table 1. Bacterial strains were taken from a 4ºC stock culture and streaked out twice on BHI (Scharlau Chemie S.A., Barcelona, Spain) agar plates for 24 hours at 37ºC. Colonies were suspended in BHI to obtain a 1 McFarland initial bacterial suspension of approximately 3×108 colony-forming units per mL (CFU/mL ). All strain cultures were checked for purity by Gram stain and colony morphology ( Table 1)

**Table 1:**
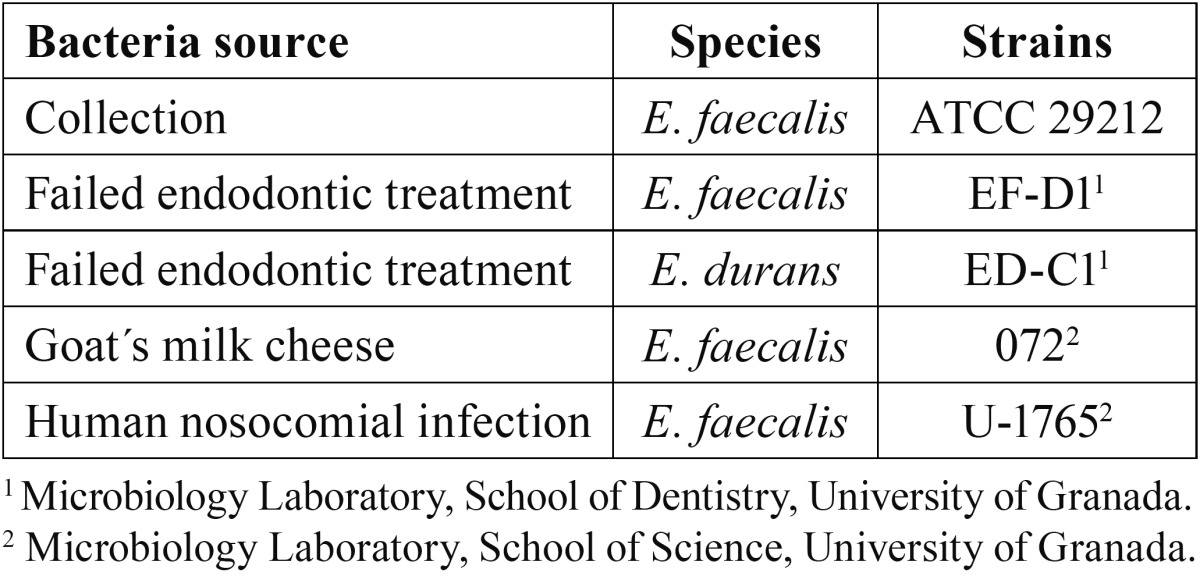
Enterococci species and strains and their source.

The CHX varnishes tested were: EC40 (35% CHX, 27% sandarac, 38% ethanol; Biodent BV, Nijmegen, The Netherlands), Cer-vitec (CE; 1% CHX, 1% thymol, ethanol, ethyl acetate, polymer; Ivoclar-Vivadent, Schaan, Liechtenstein) and Cervitec Plus (CEP; 1% CHX, 1% thymol, ethanol, water, acrylate and vinylacetate-copolymer; Ivoclar-Vivadent, Schaan, Liechtenstein.

-Direct Contact Test (DCT)

The DCT used to assess the antimicrobial activity of the CHX varnishes is based on previously reported methodology (17,18). While holding a 96-well microtiter plate (Nunclon Delta Surface; Nunc, Roskilde, Denmark) vertically, an area of established dimensions on one side of the wells was coated with an equal amount of each varnish using a cavity liner applicator. The varnishes were applied following the manufacturer´s instructions, and were allowed to dry for 30 minutes.

A 10-μL aliquot of the initial bacterial suspension was placed on the surface of each varnish. Bacterial suspensions placed on the wall of uncoated wells served as the positive control. After incubation for 1 hour at 37ºC with 95% relative humidity to ensure direct contact between bacteria and tested materials, 240 μL of sterile BHI was added to each well. The bacterial suspension was mixed for 1 minute, diluted serially and plated for viable cell counting. Ten replicates per strain and varnish were performed.

-Biofilm Formation Test (BFT)

The biofilm model used was the MBEC high-throughput (HTP) device (Innovotech, Edmonton, Alberta, Canada) (3,19,20). This batch-culture apparatus has a lid with 96 pegs that fits over a standard 96-well microtiter plate (21). Ten pegs were coated with each varnish, following the manufacturer´s instructions; and 10 uncoated pegs served as the positive and sterility controls. Each assay was performed for a total of ten replicates per strain and CHX varnish in different devices.

The wells of the microtiter plate were inoculated with 150 µL of a 1 in 30 dilution of the initial bacterial suspension, while 10 wells were inoculated with sterile BHI for the sterility control. The coated peg lid was fitted inside the wells, and the device was placed on a rocking table (Swing Sw 8 10000-00015. OVAN, Badalona, Spain) at 5 rocks per minute for 24 hours of incubation at 37ºC. Biofilms forming on the pegs were rinsed twice with 0.9% saline solution for 2 minutes to remove loosely adherent planktonic bacteria. The lid was then transferred to a microtiter recovery plate with 200 µL of BHI/well and sonicated on a water-table sonicator (Model 5510E–MT; Branson, Danbury, CT) for 10 minutes to disrupt the biofilm structure. The viability of the biofilms was determined by spot plating 10-µL aliquots of recovery biofilms onto BHI agar, then incubating for 24 hours at 37ºC.

The antimicrobial activity and the capacity to reduce biofilm formation of each CHX varnish on the strains tested were determined by calculating the percentage of reduction of viable bacteria as follows: [1-(mean CFUCHX varnish/mean CFUinitial bacterial number)] x 100. In the DCT, the term ‘eradication’ was used to denote the death of 100% of the bacterial population. The formation of enterococci biofilms was expressed as percentage of biofilm reduction with respect to the control.

To compare the efficacies of the different CHX varnishes and the strains tested when the percentage kill varied from 100%, the Student t-test was used, previously subjecting data to the Anscombe transformation.

## Results

The results of the antibacterial effects of the CHX varnishes from the DCT are listed in  Table 2. EC40 showed the best antibacterial action, eradicating all the enterococci strains except for *E. faecalis* ATCC 29212 (98.78%). CE and CEP, while variable in effectivity, produced the greatest kill percentages of strains *E. faecalis* 072 and *E. durans* ED-C1.

**Table 2:**
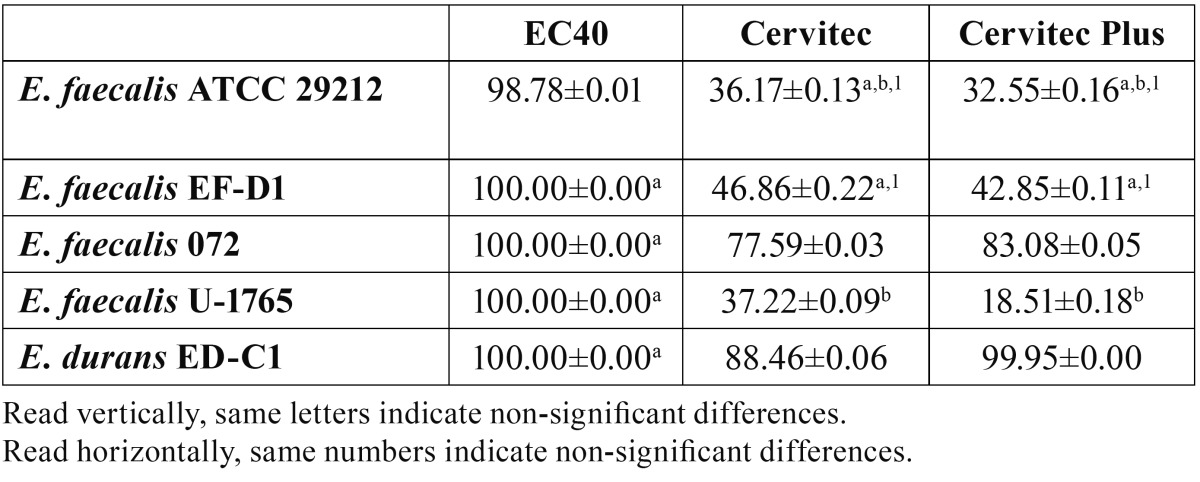
Percentage of kill of five enterococci strains after direct contact with chlorhexidine varnishes (mean±SD).

No significant differences were obtained when the effects of CE were compared with those of CEP against *E. faecalis* ATCC 29212 (p=0.549) or *E. faecalis* EF-D1 (p=0.705); but differences were indeed significant for the rest of the bacteria tested.

 Table 3 shows the percentage of reduction of biofilm formation. *E. faecalis* 072 formed no biofilm on any of the three varnishes tested (100% reduction); *E. faecalis* ATCC 29212 and *E. durans* ED-C1 were likewise unable to create biofilm upon EC40. The percentage of biofilm reduction on CE and CEP was over 92% in all cases except for *E. faecalis* U-1765 on CEP, which gave a mean value of 76.42%.

**Table 3:**
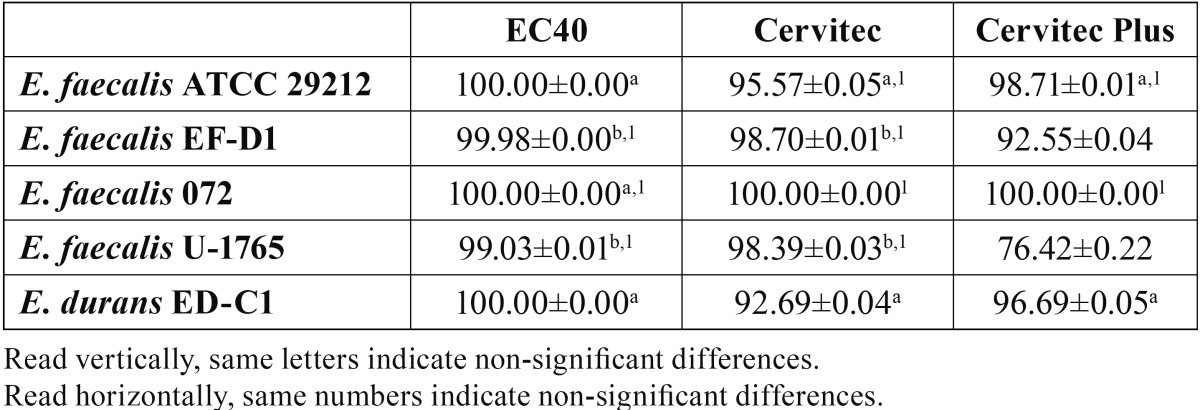
Percentage of reduction of biofilm formation of four *E. faecalis* strains and one *E. durans* strain by chlorhexidine varnishes (mean±SD).

## Discussion

The use of intracanal medication can help eliminate the bacteria remaining after chemo-mechanical instrumentation and may prevent microorganism invasion between treatment appointments (1). Our results suggest that all three varnishes studied exert antimicrobial activity against the five strains tested, and are capable of preventing or reducing the formation of biofilm.

Although the most frequently used intracanal medicament is calcium hydroxide, when CHX is used, *E. faecalis* is better eliminated from dentinal tubules (5). As inter-appointment medication, it may be applied in the form of a gel (8) or incorporated into sustained-release devices (13). The potential benefits of CHX varnishes in endodontics may stem from a greater and more prolonged release from dental tissue than with CHX gels (22), and their ability to penetrate and disinfect dentin tubules of the surface root (14).

The bacteria selected for our study were enterococci strains, particularly *E. faecalis*, which are often isolated from necrotic or improperly filled root canal systems (23). *E. faecalis* ATCC 29212 is a strain of reference widely used in antimicrobial susceptibility studies (13). Moreover, two enterococci strains isolated from failed endodontic treatments were studied, *E. faecalis* EF-D1 and *E. durans* ED-C1, the latter reportedly present in root canals (24). The origin of enterococci found in the oral cavity is unclear; and whereas the most likely source would be food (25), nosocomial infections are another possibility. We therefore used strains pertaining to both sources: *E. faecalis* 072 (26) and *E. faecalis* U-1765.

A DCT (17) with several modifications was used to evaluate the antimicrobial activity of the varnishes, since it permits a direct evaluation of their bactericidal effects. This method is quantitative and reliable, and reproduces the contact of the test microorganism with the varnishes. Our results showed that EC40 was the most effective CHX varnish, eradicating all the strains tested, except *E. faecalis* ATCC 29212. CE and CEP gave the highest kill percentages in contact with *E. durans* ED-C1 (over 88%) and *E. faecalis* 072 (over 77%), CEP proving to be significantly more effective than CE. Neither varnish produced kill over 50% against the rest of the strains. This may be due to the lesser concentration of the active ingredients in these preparations, and to the presence of polymers or copolymers in their formulation, which may limit their diffusion.

For the BFT, the MBEC-HTP device was considered appropriate because it allows for the simultaneous formation of 96 statistically equivalent biofilms under similar conditions (19,21). After 24 hours of incubation (3), the results of the positive controls showed each strain to have a different capacity for forming biofilm. *E. faecalis* 072 created a lesser amount of biofilm, which would explain its 100% reduction with the three varnishes. The two strains isolated from root canals, *E. durans* ED-C1 followed by *E. faecalis* EF-D1, formed a great amount of biofilm. This finding contrasts with the results reported by Duggan and Sedgley (27), who showed *E. faecalis* strains from endodontic sources to have a lower inherent capacity for biofilm formation.

In parallel to the results seen for DCT, EC40 was the most effective varnish, able to fully inhibit biofilm formation of three strains. This may be attributed to its high concentration of CHX, which would provide greater substantive antimicrobial activity (28). Lee et al (13) similarly obtained strong antimicrobial action on contaminated dentin blocks with *E. faecalis* using a polymeric CHX controlled device with 40% CHX. CE and CEP exhibited a lesser capacity to inhibit biofilm formation in our study, which also varied depending on the species involved. The lower concentration of CHX (1%), even though it included thymol in its composition (29), might explain these results. However, both these varnishes achieved a percentage of reduction over 92%; in contrast, CEP was not very effective against *E. faecalis* U-1765 (76.42%). There is no clear evidence pointing to one of these substances as better than the other, a point brought out previously in the context of cariogenic bacteria (30).

The study described here demonstrates that the CHX varnishes —and particularly EC40— exert effective antimicrobial action against the enterococci strains tested, and that they are able to inhibit or considerably reduce the formation of biofilms of these bacteria. Despite the limitations of this in vitro study, we may state that the results are promising, and encourage further studies to evaluate other properties of varnishes and new sustained-release systems.
